# Supplementary material for: Giant adiabatic temperature change and its direct measurement of a barocaloric effect in a charge-transfer solid
Source: Nat Commun. 2023 Dec 27;14:8466. doi: 10.1038/s41467-023-44350-4 (PMC10752886; doi:10.1038/s41467-023-44350-4)
Supplement: Supplementary file 1 — Supplementary Information [file 41467_2023_44350_MOESM1_ESM.pdf]

Giant adiabatic temperature change and its direct measurement of a barocaloric effect in a charge-transfer solid

Shin-ichi Ohkoshi,[1,2]* Kosuke Nakagawa,[1] Marie Yoshikiyo,[1] Asuka Namai,[1] Kenta Imoto,[1] Yugo Nagane,[1] Fangda Jia,[1] Olaf Stefanczyk,[1] Hiroko Tokoro,[1,3] Junhao Wang,[1,3] Takeshi Sugahara,[4] Kouji Chiba,[5] Kazuhiko Motodohi,[6] Kazuo Isogai,[6] Koki Nishioka,[6] Takashi Momiki,[6] and Ryu Hatano[6]

[1] *Department of Chemistry, School of Science, The University of Tokyo; 7-3-1 Hongo, Bunkyo-ku, Tokyo 113-0033, Japan.*
[2] *Cryogenic Research Center, The University of Tokyo; 2-11-16 Yayoi, Bunyko-ku, Tokyo 113-0032, Japan.*
[3] *Department of Materials Science, Faculty of Pure and Applied Sciences, University of Tsukuba; 1-1-1 Tennodai, Tsukuba, Ibaraki 305-8573, Japan.*
[4] *Division of Chemical Engineering, Graduate School of Engineering Science, Osaka University; 1-3 Machikaneyama, Toyonaka, Osaka 560-8531, Japan.*
[5] *Material Science Div., MOLSIS Inc.; 3-19-9 Hatchobori, Chuo-ku, Tokyo 104-0032, Japan.*
[6] *Aisin Corporation; 2-1 Asahi-machi, Kariya, Aichi 448-8650, Japan.*

*To whom correspondence should be addressed
E-mail: ohkoshi@chem.s.u-tokyo.ac.jp

Contents: **Page**

§ 1.	Nomenclature		S2
§ 2.	SEM image	Fig. 1	S4
§ 3.	Thermal durability	Fig. 2	S5
§ 4.	Crystal structure analysis of **cyano-RbMnFeCo**	Tables 1, 2<br>Figs. 3–6	S6
§ 5.	Magnetic measurements under pressure	Figs. 7–10	S11
§ 6.	Magnetic heat capacity	Fig. 11	S15
§ 7.	Justification of the present approach for entropy curves under pressure using high-pressure DSC	Figs. 12–14	S16
§ 8.	Reversible barocaloric effect in **cyano-RbMnFeCo**	Fig. 15<br>Tables 3–6	S19
§ 9.	First-principles phonon mode calculations of the entropy versus temperature	Fig. 16	S24
§ 10.	Measurement of temperature change of **cyano-RbMnFeCo**	Figs. 17, 18	S26
§ 11.	Material cost		S28
§ 12.	Devices and sensors for measurements	Table 7	S29
§ 13.	Supplementary References		S30

§ 1. Nomenclature

Symbols for each section

General symbols

T	Temperature, K
p	Pressure, Pa

Introduction

$\Delta T_{ad,rev}$	Reversible adiabatic temperature change, K
$T_{span,rev}$	Temperature window, K
ΔS_{rev}	Reversible isothermal entropy change, J K^{-1} kg^{-1}
RC_{rev}	Refrigerant capacity for reversible cycles, J kg^{-1}
dT/dp	Pressure-induced shift of the transition temperature, K GPa^{-1}
ΔT_{obs}	Temperature change directly measured by the thermocouple, K

Material and crystal structure

λ	Thermal conductivity, W m^{-1} K^{-1}

Temperature-induced phase transition due to charge transfer measured by SQUID and PXRD

χ_M	Molar magnetic susceptibility, cm^3 mol^{-1}
$T_{\downarrow}$	Temperature in the cooling process where the $\chi_M T$ change becomes half, K
$T_{\uparrow}$	Temperature in the heating process where the $\chi_M T$ change becomes half, K
β	Volume expansion coefficient, K^{-1}

Temperature-induced phase transition due to charge transfer measured by DSC

ΔH_t	Transition enthalpy, J kg^{-1}
ΔS_t	Transition entropy, J K^{-1} kg^{-1}

Pressure-induced phase transition and its reversibility

dT/dp	Pressure-induced shift of the transition temperature, K GPa^{-1}
x	Ratio of the HT phase
G	Gibbs free energy, J kg^{-1}
ΔH	Enthalpy difference between the HT and LT phases, J kg^{-1} (or J mol^{-1})
ΔS	Entropy difference between the HT and LT phases, J K^{-1} kg^{-1} (or J K^{-1} mol^{-1})
γ	Interaction parameter due to the elastic interaction, J mol^{-1}
R	Gas constant, J K^{-1} mol^{-1}

Entropy curves under ambient and high pressures

C_p	Heat capacity, J K^{-1} kg^{-1}
$C_{p,LT}$	C_p versus temperature curve for the LT phase, J K^{-1} kg^{-1}
$C_{p,HT}$	C_p versus temperature curve for the HT phase, J K^{-1} kg^{-1}
C_{mag}	Contribution from magnetic ordering on heat capacity, J K^{-1} kg^{-1}
C_{phonon}	Contribution from phonon mode (lattice vibration) on heat capacity, J K^{-1} kg^{-1}
S	Entropy, J K^{-1} kg^{-1}
$S_{LT}(T)$	Entropy versus temperature curve for the LT phase, J K^{-1} kg^{-1}
$S_{HT}(T)$	Entropy versus temperature curve for the HT phase, J K^{-1} kg^{-1}
$S_{LT}(T,p)$	Pressure-dependent entropy curve for the LT phase, J K^{-1} kg^{-1}
$S_{HT}(T,p)$	Pressure-dependent entropy curve for the HT phase, J K^{-1} kg^{-1}
σ	$\equiv (\partial S/\partial p)_T$, J K^{-1} kg^{-1} MPa^{-1}
V	Volume, m^3
T_C	Curie temperature, K

Justification of the present approach for entropy curves under pressure using high-pressure DSC

σ_{LT}	$\equiv (\partial S/\partial p)_T$ for the LT phase, J K^{-1} kg^{-1} MPa^{-1}
σ_{HT}	$\equiv (\partial S/\partial p)_T$ for the HT phase, J K^{-1} kg^{-1} MPa^{-1}
ΔS_t	Transition entropy obtained by DSC measurement, J K^{-1} kg^{-1}
Q	Heat, J
T_a	Starting temperature of the phase transition, K
T_b	Ending temperature of the phase transition, K

Giant reversible adiabatic temperature change in the barocaloric effect

$\Delta T_{ad,rev}$	Reversible adiabatic temperature change, K
ΔS_{rev}	Reversible isothermal entropy change, J K^{-1} kg^{-1}
$T_{span,rev}$	Temperature window, K
RC_{rev}	Refrigerant capacity for reversible cycles, J kg^{-1}
T_1	Starting temperature of the adiabatic pressure release process, K
T_2	Final temperature of the adiabatic pressure release process, K

First-principles phonon mode calculations of the reversible adiabatic temperature change

$S_{vib}(T)$	Vibrational entropy, J K^{-1} kg^{-1}
ΔS_{vib}	Difference between vibrational entropy of HT and LT phases, J K^{-1} kg^{-1}
S_{os}	Contribution from the orbital degeneracy and the spin multiplicity on entropy, J K^{-1} kg^{-1}
$\Delta S_{rev,calc}$	Calculated reversible isothermal entropy change from first-principles calculations, J K^{-1} kg^{-1}
$\Delta T_{ad,rev,calc}$	Calculated reversible adiabatic temperature change from first-principles calculations, K
A_{vib}	Vibrational Helmholtz energy, J mol^{-1}

Direct measurement of the temperature change using a thermocouple upon applying and releasing the pressure

ΔT_{obs}	Temperature change directly measured by the thermocouple, K

Abbreviation

HT	High-temperature
LT	Low-temperature
MF theory	Molecular field theory
SD model	Slichter-Drickamer model
CTIJT distortion	Charge-transfer–induced Jahn–Teller distortion
DSC	Differential scanning calorimeter
High-Pressure DSC	High pressure differential scanning calorimeter
PPMS	Physical properties measurement system
PXRD	Powder X-ray diffraction
TG	Thermogravimetry
SEM	Scanning electron microscopy
SQUID	Superconducting quantum interference device
VASP	Vienna *ab initio* simulation package

§ 2. SEM image

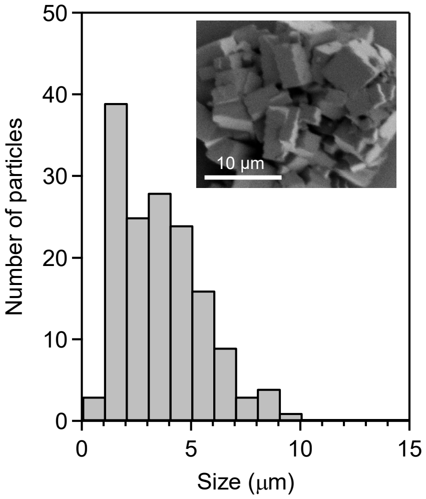

Supplementary Figure 1. SEM image and size distribution of **cyano-RbMnFeCo**.

§ 3. Thermal durability

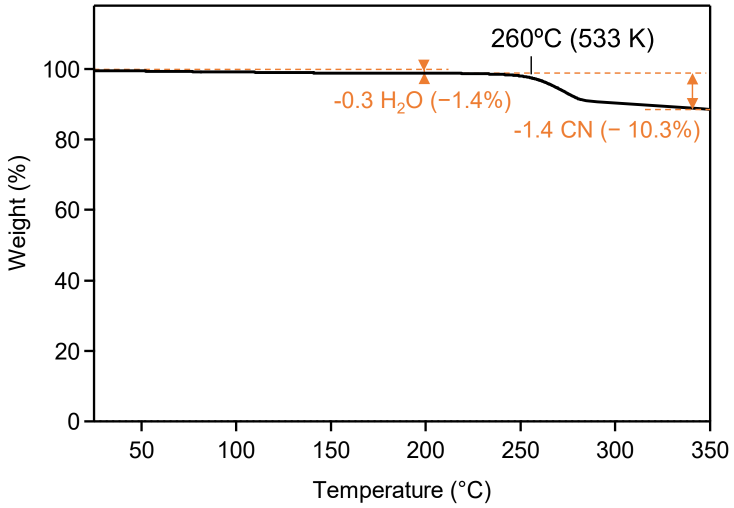

Supplementary Figure 2. Thermogravimetric curve of **cyano-RbMnFeCo** measured in air. Mass loss at 260 °C corresponds to the decomposition of the CN groups.

§ 4. Crystal structure analysis of cyano-RbMnFeCo

Supplementary Table 1. Structural details of the HT phase of **cyano-RbMnFeCo** (cubic) at 300 K obtained from the Rietveld analysis.

Formula	$C_6Co_{0.08}Fe_{0.92}H_{0.6}MnN_6O_{0.3}Rb$
Formula weight	358.01
Crystal system	Cubic
Space group	$F\bar{4}3m$ (No. 216)
a (Å)	10.5589(2)
V (Å^3)	1177.23(4)
d_{cal} (g cm^{-3})	2.00
Z	4
R_{wp} (%)	4.06
S	2.32

Atomic pos.	x/a	y/b	z/c	Occ.	multiplicity
Rb1	0.25	0.25	0.25	0.942	4
Rb2	0.25	0.25	0.75	0.058	4
Fe	0	0	0.5	0.92	4
Co	0	0	0.5	0.08	4
Mn	0	0	0	1	4
C	0	0	0.3118	1	24
N	0	0	0.2138	1	24
O1	0.25	0.25	0.75	0.06	4
O2	0.345	0.345	0.655	0.06	16

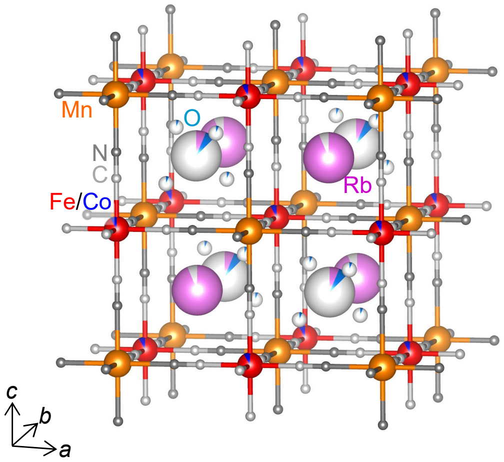

Supplementary Figure 3. Crystal structure of the HT phase of **cyano-RbMnFeCo** (cubic) at 300 K. Magenta, orange, red, blue, light gray, and gray balls represent Rb, Mn, Fe, Co, C, and N, respectively.

Supplementary Table 2. Structural details of the LT phase of **cyano-RbMnFeCo** (tetragonal) at 100 K obtained from the Rietveld analysis.

Formula	$C_6Co_{0.08}Fe_{0.92}H_{0.6}MnN_6O_{0.3}Rb$
Formula weight	358.01
Crystal system	Tetragonal
Space group	$I\bar{4}m2$ (No. 119)
a (Å)	7.1061(2)
c (Å)	10.5292(5)
V (Å^3)	531.69(4)
d_{cal} (g cm^{-3})	2.23
Z	2
R_{wp} (%)	5.12
S	2.67

Atomic pos.	x/a	y/b	z/c	Occ.	multiplicity
Rb1	0	0.5	0.25	0.942	2
Rb2	0	0.5	0.75	0.058	2
Fe	0	0	0	0.92	2
Co	0	0	0	0.08	2
Mn	0	0	0.5	1	2
C1	0	0	0.1738	1	4
N1	0	0	0.2847	1	4
C2	0.1882	0.1882	0	1	8
N2	0.3014	0.3014	0	1	8
O1	0	0.5	0.75	0.06	2
O2	0.306	0	0.153	0.06	8

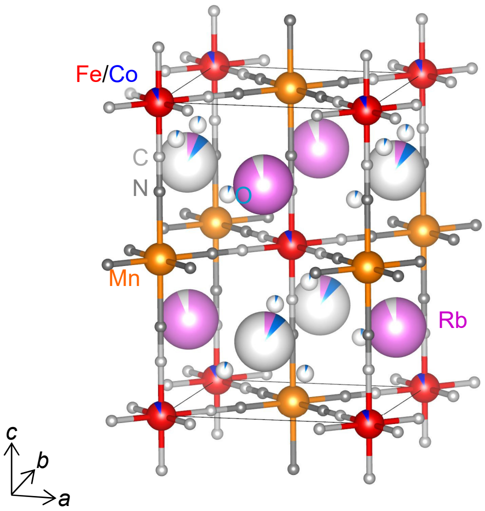

Supplementary Figure 4. Crystal structure of the LT phase of **cyano-RbMnFeCo** (tetragonal) at 100 K. Magenta, orange, red, blue, light gray, and gray balls represent Rb, Mn, Fe, Co, C, and N, respectively.

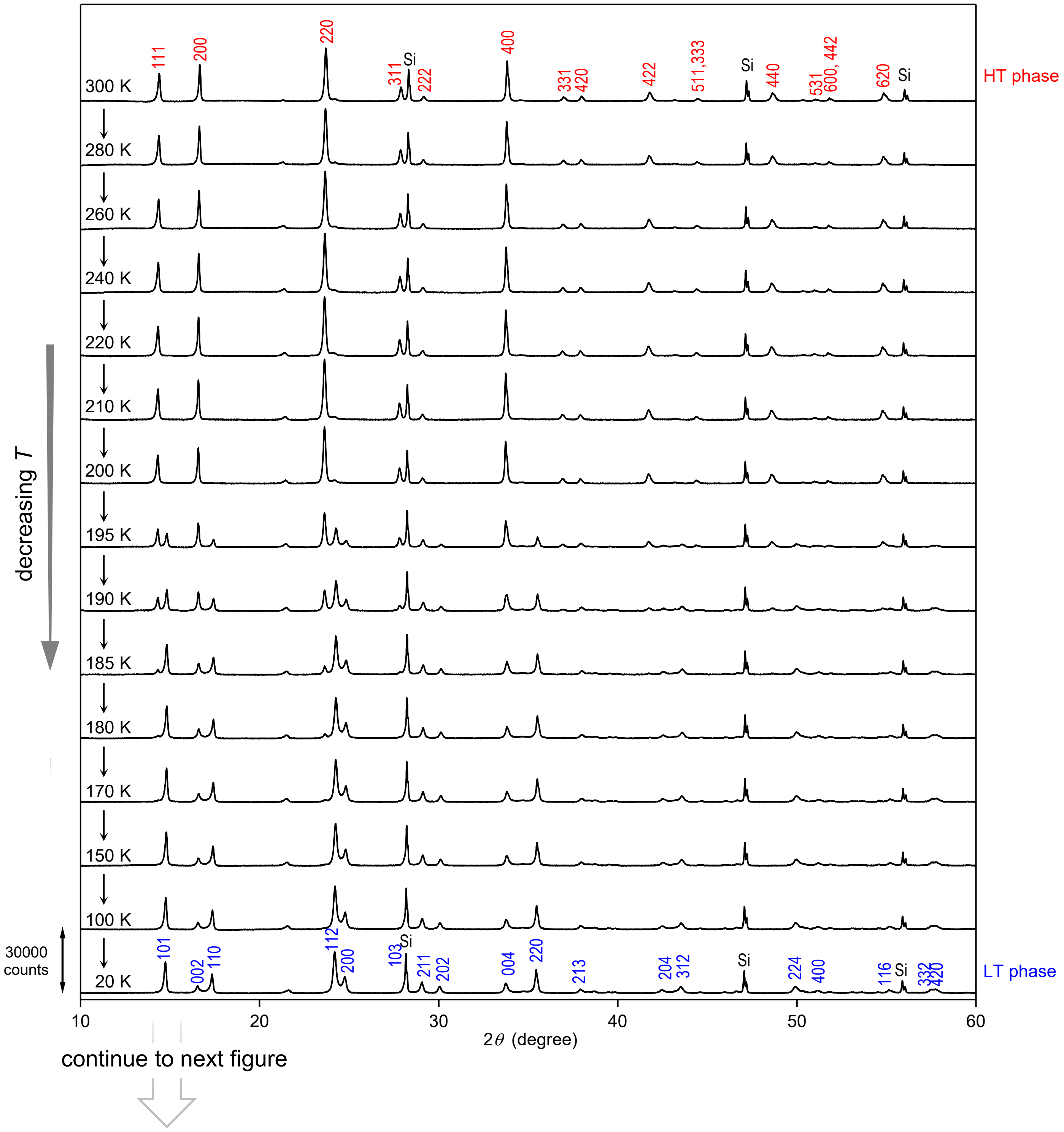
111
200
220
311
Si
222
400
331
420
422
511,333
Si
440
531
600, 442
620
Si
HT phase
300 K
280 K
260 K
240 K
220 K
210 K
200 K
195 K
190 K
185 K
180 K
170 K
150 K
100 K
20 K
decreasing T
30000 counts
101
002
110
112
200
103
Si
211
202
004
220
213
204
312
Si
224
400
116
Si
332
420
LT phase
10
20
30
40
50
60
2θ (degree)
continue to next figure

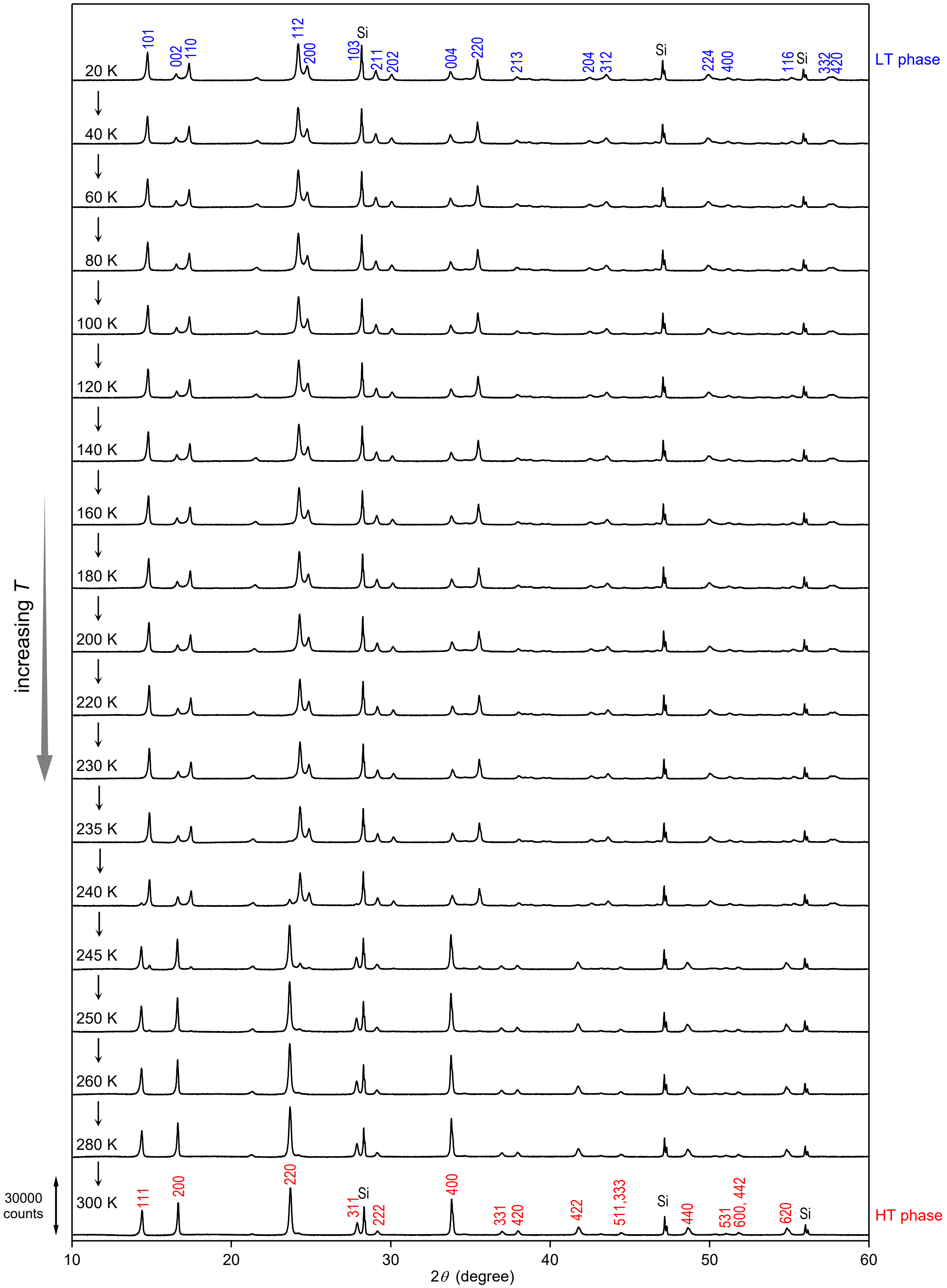

Supplementary Figure 5. Temperature dependence of the PXRD patterns of cyano-RbMnFeCo. Red and blue numbers correspond to the indices for the HT and LT phases, respectively.

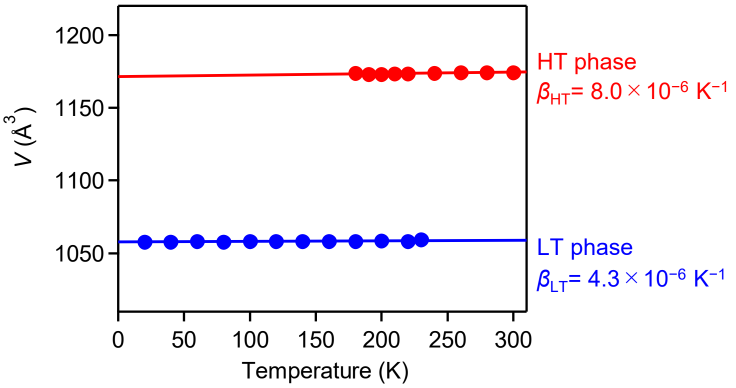

Supplementary Figure 6. Temperature dependence of the lattice volume. Temperature dependence of the lattice volume of the HT phase (red) and twice the value of the lattice volume for the LT phase (blue) obtained by Rietveld analyses of the PXRD patterns at ambient pressure. Note that the number of atoms in the unit cell of the HT phase is twice that of the LT phase due to the difference in the crystallographic symmetry. β_{HT} and β_{LT} are the volume expansion coefficients of the HT and LT phases, respectively.

§ 5. Magnetic measurements under pressure

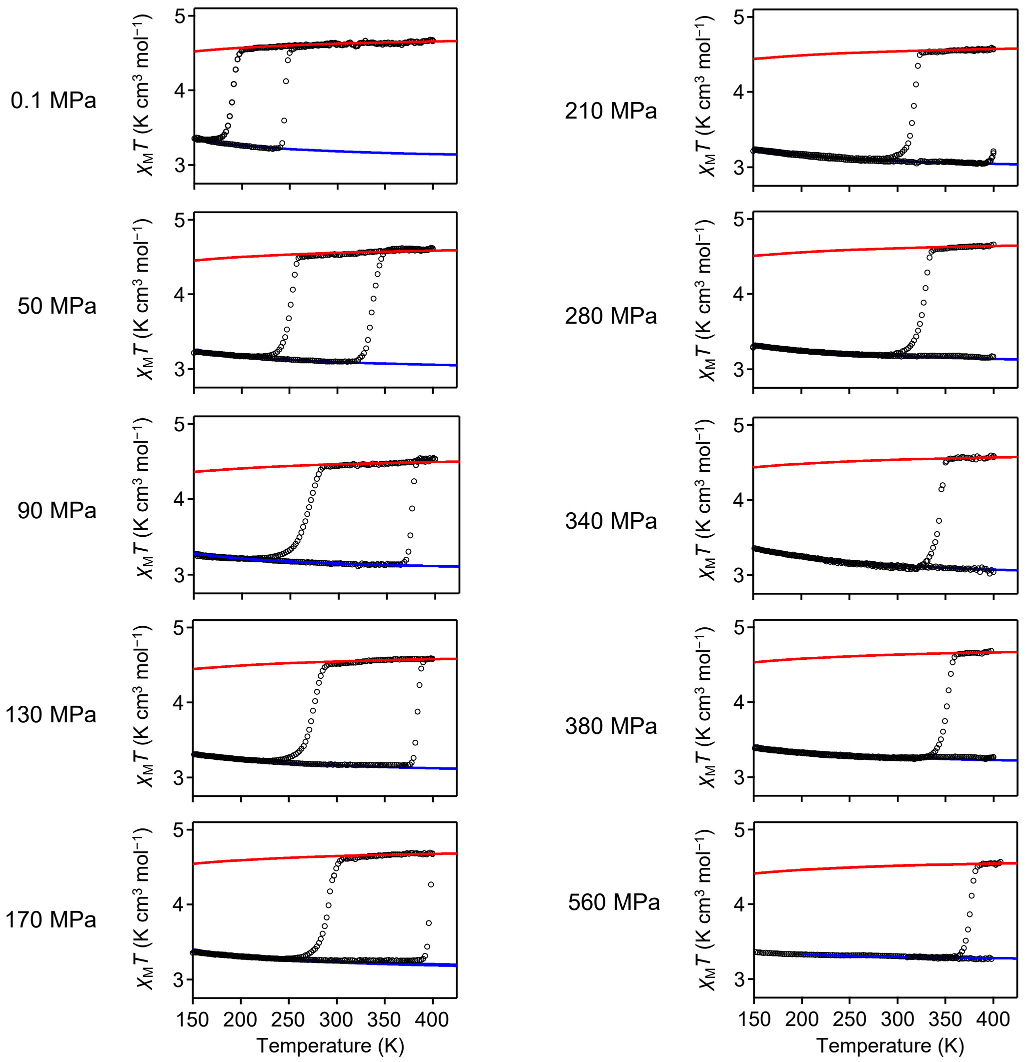

Supplementary Figure 7. $\chi_M T$ versus T plots at various pressures. $\chi_M T$ value of the HT phase is fitted using a molecular field model containing $Mn^{II}(^6A_{1g}, S = 5/2)$ and $Fe^{III}(^2T_{2g}, S = 1/2)$ with a sum of the ferromagnetic superexchange interaction of 0.5 cm^{-1} and the antiferromagnetic superexchange interaction of −2.0 cm^{-1} (red lines), while that of the LT phase is fitted with a molecular field model containing $Mn^{III}(^5B_{1g}, S = 2)$ and the ferromagnetic superexchange interaction of 0.5 cm^{-1} (blue lines). For measurements at pressures of 280–560 MPa, the sample was heated above 400 K using a lab-made heater because the upper temperature limit of the SQUID magnetometer was 400 K. First, the pressure cell containing **cyano-RbMnFeCo** was put in a glass tube together with a thermometer. Then it was heated in an oil bath. Once the desired temperature was reached, the pressure cell was transferred from the lab-made heater to the SQUID device in 20 s. The decay of the temperature versus time plot during this transfer showed an exponential decrease of $T = 433 + 39\exp(-t/30)$. Here, t is time in seconds. When the sample cooled down to 400 K in the SQUID magnetometer set to 400 K, the magnetic measurement started.

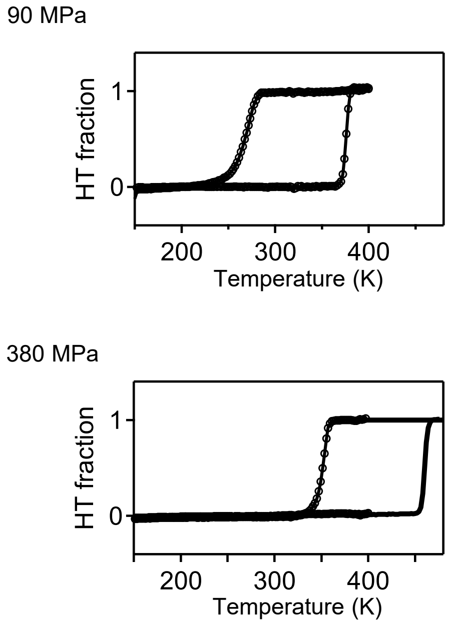

Supplementary Figure 8. Thermal hystereses of the HT phase fraction versus temperature at 90 MPa and 380 MPa. The hystereses are obtained from the experimentally obtained $\chi_M T$–T plots.

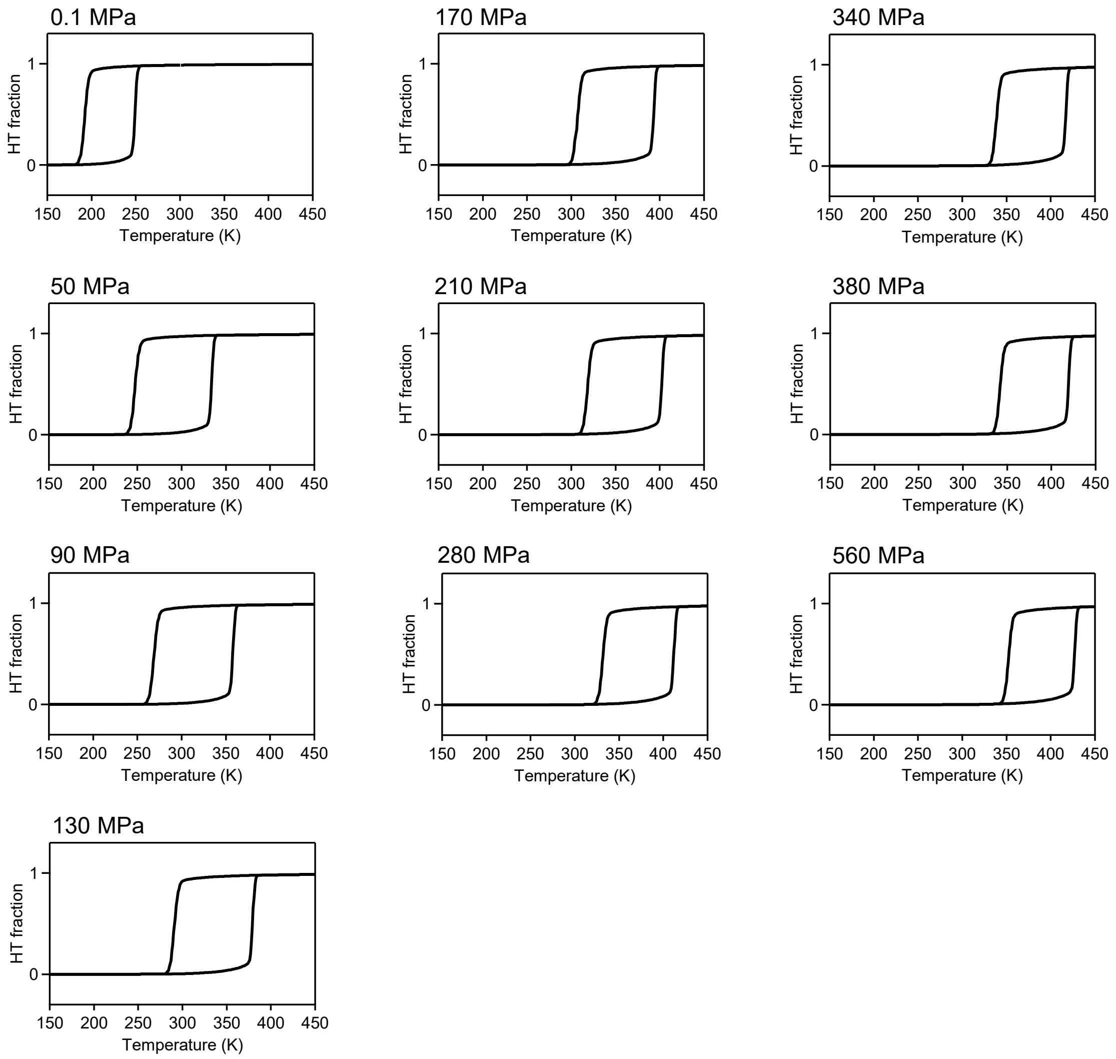

Supplementary Figure 9. HT fraction versus temperature plots calculated by the SD model. Applied pressures are 0.1 MPa, 50 MPa, 90 MPa, 130 MPa, 170 MPa, 210 MPa, 280 MPa, 340 MPa, 380 MPa, and 560 MPa. The simulation parameters are shown in the Methods section.

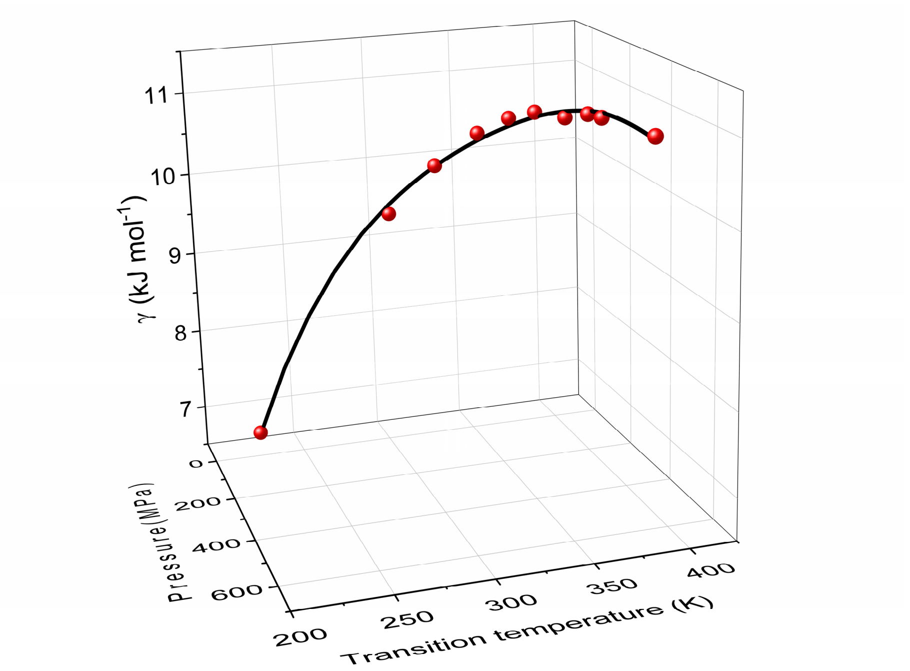

Supplementary Figure 10. Pressure and temperature dependence of the interaction parameter (γ). The γ parameter in the SD model is interpreted as the interface stress between the HT and LT phase domains inside the crystal. The γ value is known to depend on both the temperature and the pressure[48]. The temperature-dependent term $\gamma(T)$ is assumed here as $\gamma(T) = \gamma_{T,0} + \gamma_{T,1}\exp(aT)$. The pressure-dependent term $\gamma(p)$ is expressed as $\gamma(p) = \gamma_{p,1}p + \gamma_{p,2}p^2$. Thus, γ is $\gamma(T, p) = \gamma_{T,0} + \gamma_{T,1}\exp(aT) + \gamma_{p,1}p + \gamma_{p,2}p^2$. The plot of the γ values from the SD model simulations (red circles) is well reproduced by the parameters of $\gamma_{T,0}$ = 10.7(1) kJ mol^{-1}, $\gamma_{T,1} = -1.2(5)\times 10^2$ kJ mol^{-1}, $a = -1.5(2)\times 10^{-2}$ K^{-1}, $\gamma_{p,1} = 2.9(5)\times 10^{-3}$ kJ mol^{-1} MPa^{-1}, and $\gamma_{p,2} = -4.0(9)\times 10^{-6}$ kJ mol^{-1} MPa^{-2} (black line). One of the reasons for the changes of the thermal hysteresis is considered as follows. The transition temperature shifts to higher temperatures upon applying external pressure, increasing the volume difference between the HT and LT phases. The increase in the volume difference induces a larger mismatch between the HT and LT phase domains, which is expected to enhance the surface stress at the domain interface.

§ 6. Magnetic heat capacity

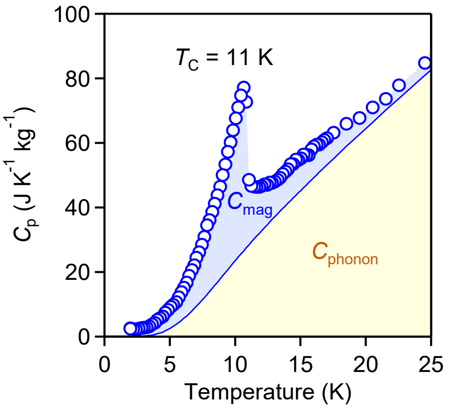

Supplementary Figure 11. Magnetic heat capacity. As the temperature increases, the C_p value monotonically increases, and a peak is observed at 11 K. C_p value is described as the sum of the contributions from the phonon mode (lattice vibration), C_{phonon}, and magnetic ordering, C_{mag} : $C_p = C_{phonon} + C_{mag}$. Blue line shows the C_{phonon} curve from first-principles phonon mode calculation. C_{mag} is obtained by subtracting C_{phonon} from C_p.

§ 7. Justification of the present approach for entropy curves under pressure using high-pressure DSC

Justification of the treatment to evaluate the pressure-dependent entropy curves in the present work was proved by heat-capacity measurements of a reference compound using a high-pressure differential scanning calorimeter (high-pressure DSC). Due to the limited measurement conditions (temperature and pressure) of our high-pressure DSC apparatus (Micro DSC 7 evo of SETARAM), an analogous compound $Rb_{0.994}Mn[Fe(CN)_6]_{0.998}\cdot 0.6H_2O$ (sample A) was used.

Firstly, the entropy curves of sample A at p = 1 bar (0.1 MPa) were determined. The entropy curve of the LT phase for sample A was evaluated from the heat capacity (C_p) measurement using PPMS, and then the transition entropy curve from the DSC measurement was added. After that, the entropy curve of the HT phase from the C_p measurement of sample A was connected. Secondly, based on these entropy curves, we evaluated the pressure-dependent entropy curves using the Maxwell relation $(\partial S/\partial p)_T = -(\partial V/\partial T)_p$. The $(\partial S/\partial p)_T$ ($\equiv \sigma$) values for the HT and LT phases of sample A were determined from the variable temperature PXRD (VT-PXRD) measurements as $\sigma_{HT} = -1.7\times10^{-3}$ J K^{-1} kg^{-1} MPa^{-1} and $\sigma_{LT} = 1.3\times10^{-3}$ J K^{-1} kg^{-1} MPa^{-1}, respectively. (These σ values are very small compared to polymers or plastic crystals because the present material is an inorganic material exhibiting zero thermal expansion.) Assuming that these σ values are independent of pressure, the entropy curves under pressure were obtained. As an example, the entropy curves of the LT and HT phases at 85 MPa (0.85 kbar) are shown in Supplementary Fig. 12. In the present analysis method, the HT phase fraction (x), which is calculated from the magnetic susceptibility measurements, is used to connect the LT and HT entropy curves (black dotted line in Supplementary Fig. 12b). When the phase transition temperature is 331 K, the ΔS value is estimated to be 190 J K^{-1} kg^{-1} (Supplementary Fig. 12b).

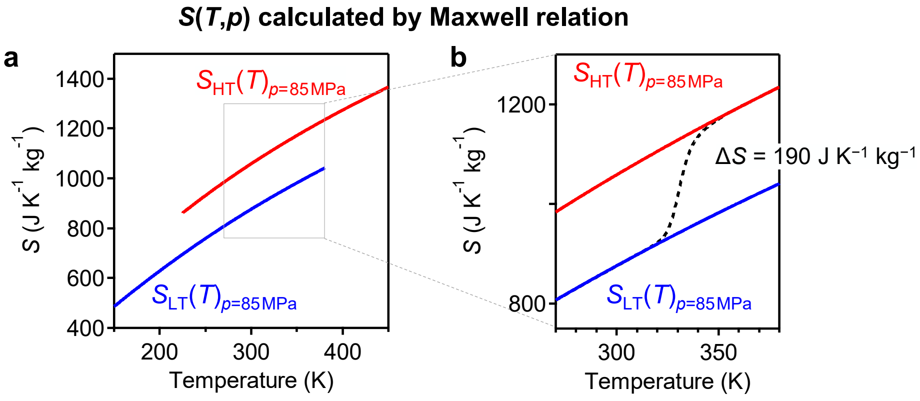

Supplementary Figure 12. Process to obtain the entropy curves at 85 MPa (0.85 kbar). **a**, Entropy curves of sample A at 85 MPa (0.85 kbar) for the LT phase (blue) and the HT phase (red) calculated based on the Maxwell relation. **b**, Enlarged entropy curves of the LT and HT phases at 85 MPa, connected by the entropy curve based on the HT phase fraction x calculated from the magnetic measurement at 0.1 MPa.

To confirm the accuracy of the estimated entropy curves under pressure, a high-pressure DSC measurement was conducted at p= 85 MPa (the upper pressure limit of this apparatus is 100 MPa). The result shows an endothermic peak with a transition entropy of ΔS_t = 186 J K^{-1} kg^{-1} at 331 K (Supplementary Fig. 13a). The entropy curve from the high-pressure DSC was connected to the $S_{LT}(T)_{p=85MPa}$ curve using $S(T,p) = S(T_a, p) + \int_{T_a}^{T} 1/T\,(C_p + dQ/dT)\,dT$ ($T_a < T \leq T_b$), where dQ/dT is the heat flow, and T_a and T_b are the starting and ending temperatures of the phase transition in the high-pressure DSC measurement, respectively (Supplementary Fig. 13b). Considering the contribution from the C_p term, the ΔS value is obtained as 189 J K^{-1} kg^{-1}, which is consistent with ΔS= 190 J K^{-1} kg^{-1} estimated from the Maxwell relation and the x vs. T curve from SQUID. The difference is less than 1% (Supplementary Fig. 13b, right). This good agreement clearly shows that the present analysis method can accurately provide entropy curves under pressure.

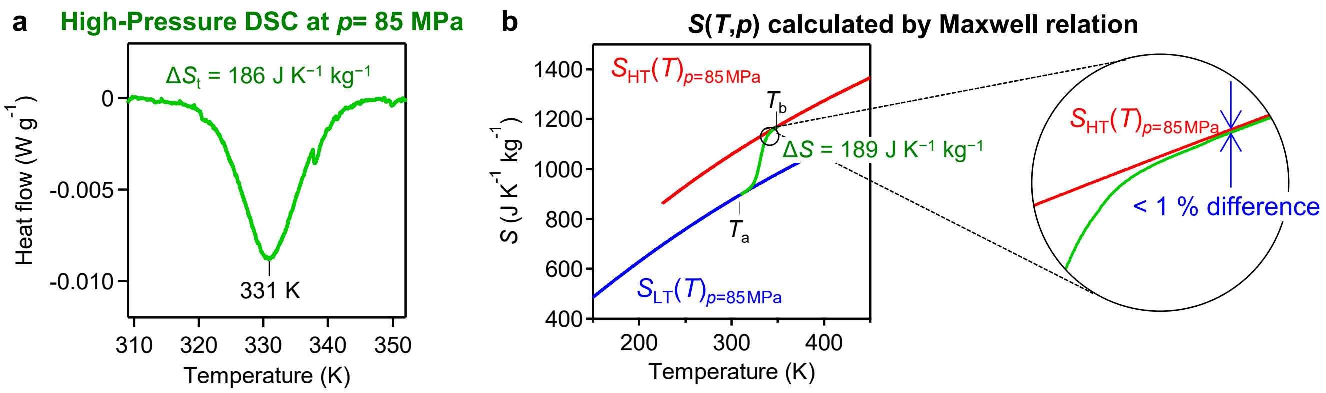

Supplementary Figure 13. Process to obtain entropy curve based on a high-pressure DSC measurement. a, Heat flow vs. T curve of sample A at 85 MPa (0.85 kbar) in the heating process from a high-pressure DSC measurement showing an endothermic peak at 331 K. **b**, Entropy at 85 MPa for the LT (blue) and HT (red) phases calculated based on the Maxwell relation. Green line indicates the entropy curve obtained by integrating the high-pressure DSC curve at 85 MPa, which approaches the $S_{HT}(T)_{p=85MPa}$ curve calculated by the Maxwell relation with only a slight gap of less than 1% (right).

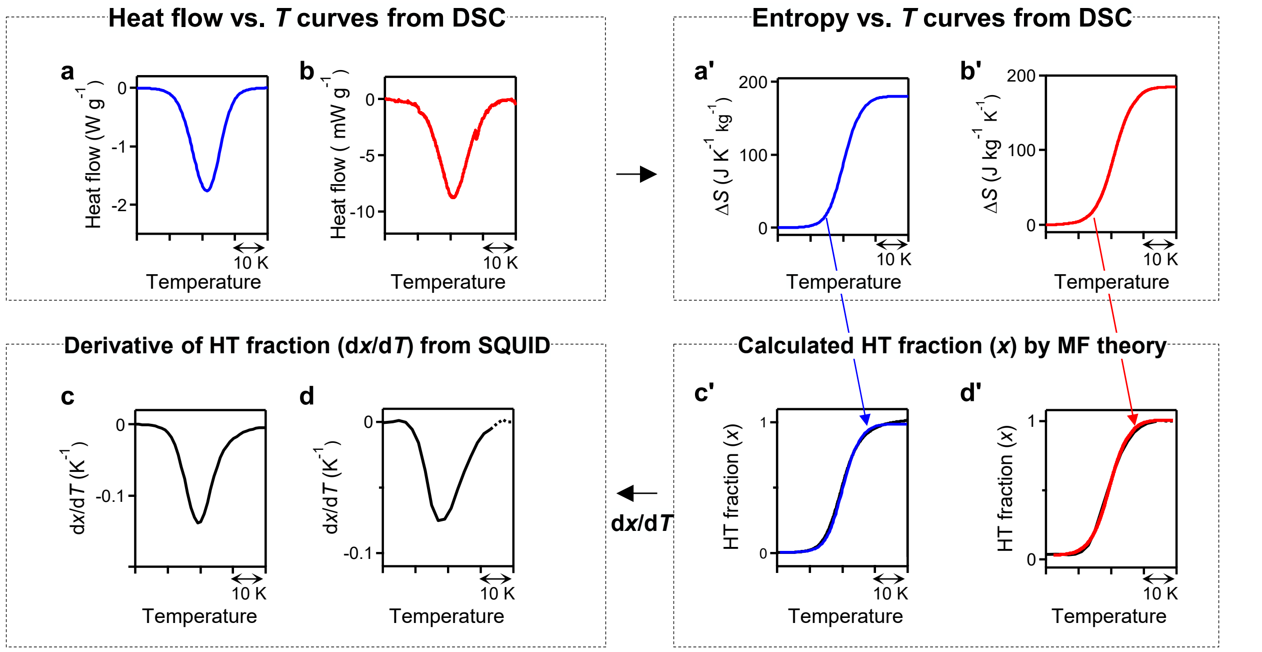

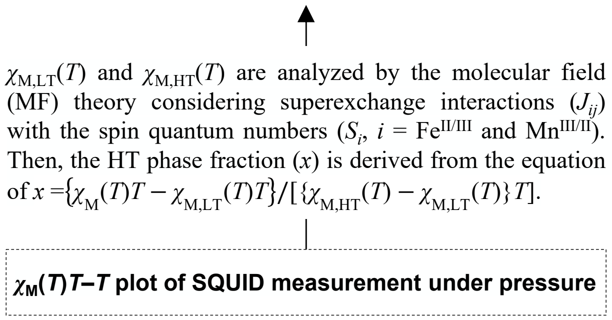

Supplementary Figure 14. The curvature of the entropy at the phase transition temperature under pressure from the SQUID data. Heat flow vs. T curve of sample A at (**a**) 0.1 MPa and (**b**) 85 MPa (0.85 kbar) in the heating process from DSC measurements. The transition entropy curves converted from (**a**) and (**b**) are shown in (**a'**) and (**b'**), respectively. Temperature derivative of HT fraction (dx/dT) vs temperature at (**c**) 0.1 MPa and (**d**) 85 MPa (0.85 kbar), which are converted from the x vs. T curves in (**c'**) and (**d'**), respectively.

Furthermore, the curvature of the entropy at the phase transition temperature under pressure from the SQUID data was validated by heat-capacity measurements. In the present work, $\chi_{M,LT}(T)$ and $\chi_{M,HT}(T)$, which depend on temperature, are analyzed by the MF theory considering superexchange interactions (J_{ij}) with the spin quantum numbers (S_i, i = $Fe^{II/III}$ and $Mn^{III/II}$). Then, the HT phase fraction x is obtained from the equation of $x = \{\chi_{M}(T)T - \chi_{M,LT}(T)T\} / [\{\chi_{M,HT}(T) - \chi_{M,LT}(T)\}T]$. In Supplementary Fig. 14, **a'** and **b'** show the transition entropy curves converted from the heat flow vs. T curves in **a** and b, respectively. Moreover, **c'** and **d'** show the x vs. T curves from the above equation concerning x. **c** and **d** were converted by dx/dT from the x vs. T curves in **c'** and **d'**, respectively. The nearly identical curvatures indicate that the HT phase fraction x, analyzed from the magnetic susceptibility, can be regarded as the phase fraction in the entropy curve between the LT and HT phases.

§ 8. Reversible barocaloric effect in cyano-RbMnFeCo

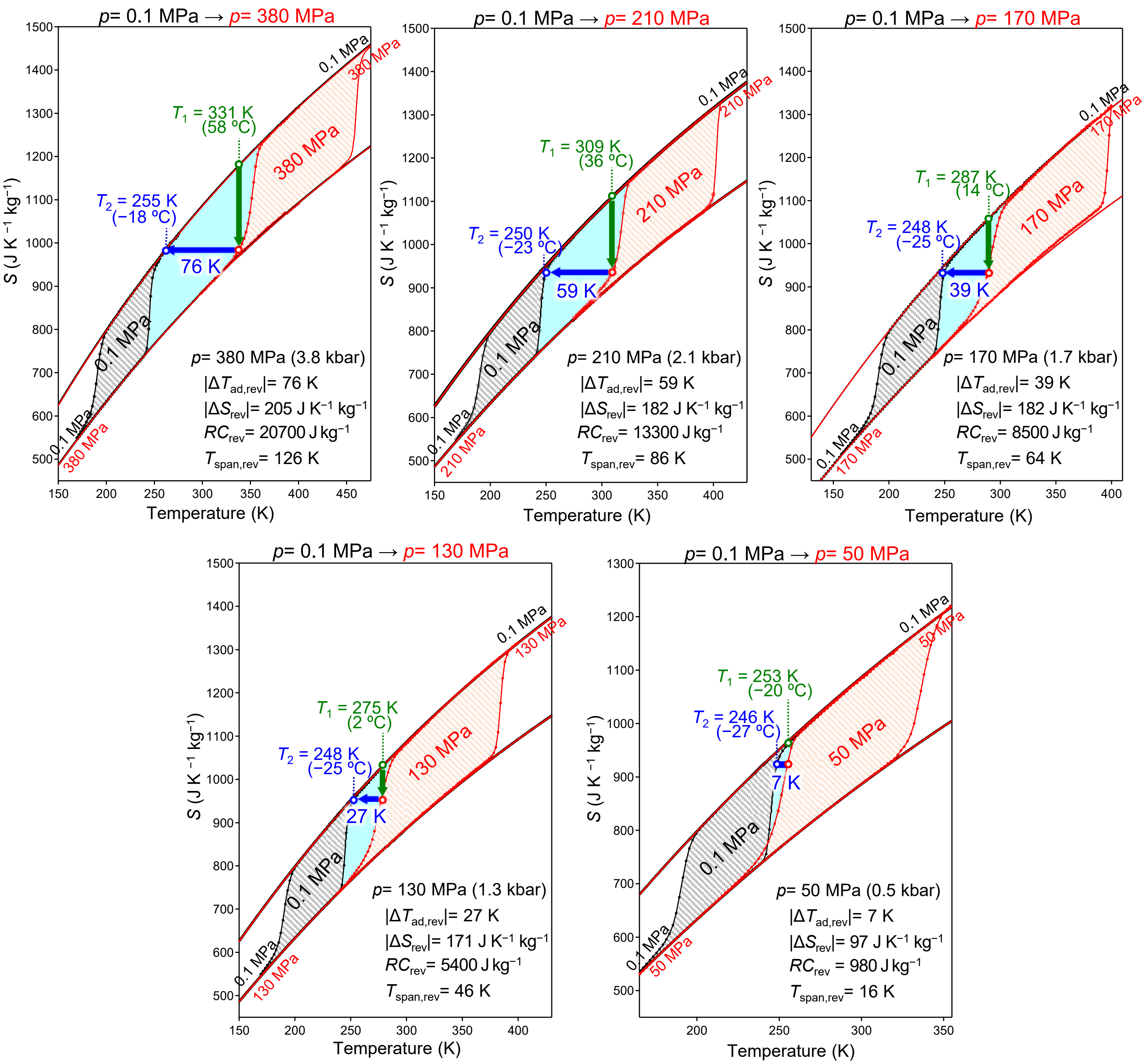

Supplementary Figure 15. Evaluation of the reversible barocaloric effect in cyano-RbMnFeCo. Entropy versus temperature curves of the HT phase and LT phase of **cyano-RbMnFeCo** obtained from the heat capacity measurements using PPMS and DSC. Black and orange shaded areas indicate the thermal hysteresis loops at 0.1 MPa and upon applying pressure (380 MPa, 210 MPa, 170 MPa, 130 MPa, and 50 MPa), respectively.

Supplementary Table 3. Reversible adiabatic temperature change ($|\Delta T_{\mathbf{ad,rev}}|$) of **cyano-RbMnFeCo** (this work) compared with those of representative solid-state barocaloric materials around room temperature.

Materials	$\|\Delta T_{\mathbf{ad,rev}}\|$ (K)	p (MPa)	Starting → final temperature (K)	Notes
cyano-RbMnFeCo	85	560	361 → 276	This work
cyano-RbMnFeCo	74	340	330 → 256	This work
cyano-RbMnFeCo	64	280	315 → 251	This work
1-Br-adamantane	55	240	383 → 328	[1]
Neopentyl alcohol (NPA)	42	580	277 → 235	[2]
$Fe_3(bntrz)_6(tcnset)_6$	35	260	375 → 340	[3]
NH_4I	34	80	298 → 264	[4]
$(C_{10}H_{21}NH_3)_2MnCl_4$	32	250	345 → 313	[5]
Neopentyl glycol (NPG)	30	570	348 → 318	[6]
Polydimethylsiloxane	28.5	390	283 → 254.5	[7]
Neopentyl glycol (NPG)	24	590	342 → 318	[2]
cyano-RbMnFeCo	21	90	269 → 248	This work
$N(CH_3)_4[FeCl_4]$	21	90	410 → 389	[8]
Acetoxy silicone rubber	20.4	173	245 → 224.6	[9]
AgI	18	250	390 → 372	[10]
Neopentyl alcohol (NPA)	16	260	251 → 235	[2]
C_{60}	16	410	278 → 262	[11]
$(C_{10}H_{21}NH_3)_2MnCl_4$	12	100	324 → 312	[5]
C_{60}	9.7	100	269.5 → 259.8	[11]
$(C_{10}H_{21}NH_3)_2MnCl_4$	7	50	317 → 310	[12]

[1] Read from Fig. 6f.[1]
[2] Read from the text on page 645 for NPA, and Fig. 6e for NPG.[2]
[3] Read from the text on page 6.[3]
[4] Read from Supplementary Fig. 3 and Fig. 4.[4]
[5] Read from Fig. 5b and the text on page 6.[5]
[6] Read from the text on page 5.[6]
[7] Read from Table 2.[7]
[8] Read from Fig. 9d and the text on page 8.[8]
[9] Read from the text on page 1003.[9]
[10] Read from the text on page 3.[10]
[11] Read from Table 1.[11]
[12] Read from the text on page 6.[12]

Supplementary Table 4. Reversible entropy change ($|\Delta \boldsymbol{S}_{\mathbf{rev}}|$) of **cyano-RbMnFeCo** (this work) compared with those of representative solid-state barocaloric materials around room temperature.

Materials	$|\Delta \boldsymbol{S}_{\mathbf{rev}}|$ (J K^{-1} kg^{-1})	p (MPa)	Starting temperature (K)	Notes
Neopentyl glycol (NPG)	510	590	330	[1], [2]
Pentaglycerol (PG)	490	240	366	[2]
Neopentyl alcohol (NPA)	470	580	274	[2]
$(C_{10}H_{21}NH_3)_2MnCl_4$	250	100	315	[3]
$(C_{10}H_{21}NH_3)_2MnCl_4$	248	50	309	[4]
1-Br-adamantane	223	240	384	[5]
cyano-RbMnFeCo	205	380	323	This work
cyano-RbMnFeCo	195	280	303	This work
Acetoxy silicone rubber	182	173	250	[6]
cyano-RbMnFeCo	150	90	251	This work
Polydimethylsiloxane rubber	121	390	323	[7]
Fe_3(bntrz)$_6$(tcnset)$_6$	120	260	375	[8]
NH_4I	71	80	308	[9]
AgI	60	250	398	[10]
C_{60}	42	410	257	[11]
C_{60}	32	100	257	[11]

[1] Read from the text on pages 4 and 5.[6]
[2] Read from Fig. 6d for NPG, the text on page 645 for PG and NPA.[2]
[3] Read from the text on page 6.[5]
[4] Read from the text on page 6.[12]
[5] Read from Fig. 6e.[1]
[6] Read from the text on page 1003.[9]
[7] Read from Table 2.[7]
[8] Read from the text on page 6.[3]
[9] Estimated from Supplementary Fig. 3h.[4]
[10] Read from the text on page 3.[10]
[11] Read from Table 1.[11]

Supplementary Table 5. Refrigerant capacity for reversible cycles ($\boldsymbol{RC}_{\mathbf{rev}}$) of **cyano-RbMnFeCo** (this work) compared with those of representative solid-state barocaloric materials.

Materials	$\boldsymbol{RC}_{\mathbf{rev}}$ (J kg^{-1})	p (MPa)	Notes
cyano-RbMnFeCo	26000	560	This work
Neopentyl alcohol (NPA)	23000	580	[1]
cyano-RbMnFeCo	20700	380	This work
Neopentyl alcohol (NPA)	17000	510	[1]
cyano-RbMnFeCo	15600	280	This work
1-Br-adamantane	14500	240	[2]
Neopentyl glycol (NPG)	13000	590	[1]
$(C_{10}H_{21}NH_3)_2MnCl_4$	9000	250	[3]
$(N(CH_3)_4[FeCl_4])$	8900	220	[4]
$Fe_3(bntrz)_6(tcnset)_6$	5800	200	[5]
Pentaglycerol (PG)	5000	240	[1]
C_{60}	3800	590	[6]
cyano-RbMnFeCo	3700	90	This work
$(C_{10}H_{21}NH_3)_2MnCl_4$	3500	100	[3]
NH_4I	3200	80	[7]
Acetoxy silicone rubber	2600	173	[8]
Polydimethylsiloxane	1900	200	[9]
$(C_{10}H_{21}NH_3)_2MnCl_4$	1400	50	[10]

[1] Read from Fig. 6f.[2]
[2] Read from Fig. 6g.[1]
[3] Read from Fig. 5c and the text on page 6.[5]
[4] Estimated from Fig. 9c.[8]
[5] Read from the text on page 6.[3]
[6] Read from Fig. 6c.[11]
[7] Estimated from Supplementary Fig. 3h.[4]
[8] Estimated from the product of normalized refrigerant capacity of ~15 kJ kg^{-1} GPa^{-1} and $|\Delta p|$ = 173 MPa given on page 1003.[9]
[9] Estimated from the product of normalized refrigerant capacity of 9.3 kJ kg^{-1} GPa^{-1} and $|\Delta p|$ = 200 MPa given in Table 4.[7]
[10] Read from Fig. 5c.[12]

Supplementary Table 6. Temperature window ($\boldsymbol{T}_{\mathbf{span,rev}}$) of **cyano-RbMnFeCo** (this work) compared with those of representative solid-state barocaloric materials.

Materials	$\boldsymbol{T}_{\mathbf{span,rev}}$ (K)	p (MPa)	Notes
cyano-RbMnFeCo	142	560	This work
cyano-RbMnFeCo	126	380	This work
cyano-RbMnFeCo	102	280	This work
C_{60}	96	590	[1]
Acetoxy silicone rubber	90	173	[2]
$(N(CH_3)_4[FeCl_4])$	82	220	[3]
1-Br-adamantane	75	240	[4]
$Fe_3(bntrz)_6(tcnset)_6$	65	260	[5]
NH_4I	51	80	[6]
$(C_{10}H_{21}NH_3)_2MnCl_4$	46	250	[7]
Neopentyl alcohol (NPA)	45	580	[8]
cyano-RbMnFeCo	38	90	This work
Neopentyl glycol (NPG)	28	570	[9]
AgI	27	250	[10]
$(C_{10}H_{21}NH_3)_2MnCl_4$	12	50	[11]

[1] Read from Fig. 5a.[11]
[2] Read from Fig. 3c.[9]
[3] Read from Fig. 9c.[8]
[4] Read from Fig. 4c.[1]
[5] Read from Fig. 6a.[3]
[6] Estimated from Supplementary Fig. 3h.[4]
[7] Read from Fig. 5a.[5]
[8] Read from Fig. 4d.[2]
[9] Read from Fig. 3c.[6]
[10] Read from Fig. 3c.[10]
[11] Read from Fig. 5b.[12]

§ 9. First-principles phonon mode calculations of the entropy versus temperature

First-principles phonon mode calculations based on density functional theory were conducted for rubidium manganese hexacyanoferrate, $RbMn[Fe(CN)_6]$, using the MedeA Phonon code by GGA + U/PBE [13–19]. Wave functions based on the plane waves and the potentials of the core orbitals were represented by the projector-augmented wave of Blöchl. The exchange-correlation term was evaluated by the generalized gradient approximation by Perdew, Burke, and Ernzerhof. The reported tetragonal and cubic lattice parameters for the LT and HT phases of rubidium manganese hexacyanoferrate were used as the initial structures in the computed models. The lattice parameters and atomic positions were optimized with an energy cutoff of 500 eV and a $3 \times 3 \times 3$ *k*-mesh until the 10^{-5} eV pm^{-1} force tolerance was satisfied using the Vienna *ab initio* simulation package (MedeA VASP). The optimized structures of the $\sqrt{2} \times \sqrt{2} \times 1$ supercells were used to calculate the phonon modes of $RbMn[Fe(CN)_6]$. The phonon modes were determined by the direct method implemented in the Phonon code with 2-pm displacements using the optimized atomic positions. The U–J value was set to 4.0 eV for Fe and Mn. It should be noted that the calculated Raman and IR frequencies agree well with the experimental spectra, indicating that the phonon mode calculation results are reasonable.

To account for the effect of thermal expansion on the entropy versus temperature curve, the thermal expansion coefficient was calculated under the quasi-harmonic approximation. The structure optimization and phonon mode calculations of the thermodynamic parameters were calculated at nine different pressures, +2.0, +1.5, +1.0, +0.5, 0, −0.5, −1.0, −1.5, and −2.0 GPa. The vibrational Helmholtz energy ($A_{\mathrm{vib},i}$) for temperatures between 0 K and 500 K was plotted against the optimized cell volume (Fig. S11). The volume with the minimum Helmholtz energy was determined for each temperature. Then the temperature expansion coefficients were obtained for the LT and HT phases. The $S_{\mathrm{vib},i}(T)$ for each temperature was taken from the entropy value at the volume with the minimum Helmholtz energy. For the calculation of the $S_{\mathrm{vib},i}(T)$ values at p = 280 MPa, the optimized cell volume at 280 MPa and 0 K, and the same temperature expansion coefficient were used.

Furthermore, the pressure effect on the transition temperature was investigated. To compare the energies between the HT and LT phases, the enthalpy values at 0 K for the two phases were calculated using a screened Coulomb hybrid functional based on the Heyd–Scuseria–Ernzerhof (HSE06) hybrid functional. The calculation results support the large dT/dp value of the present material.

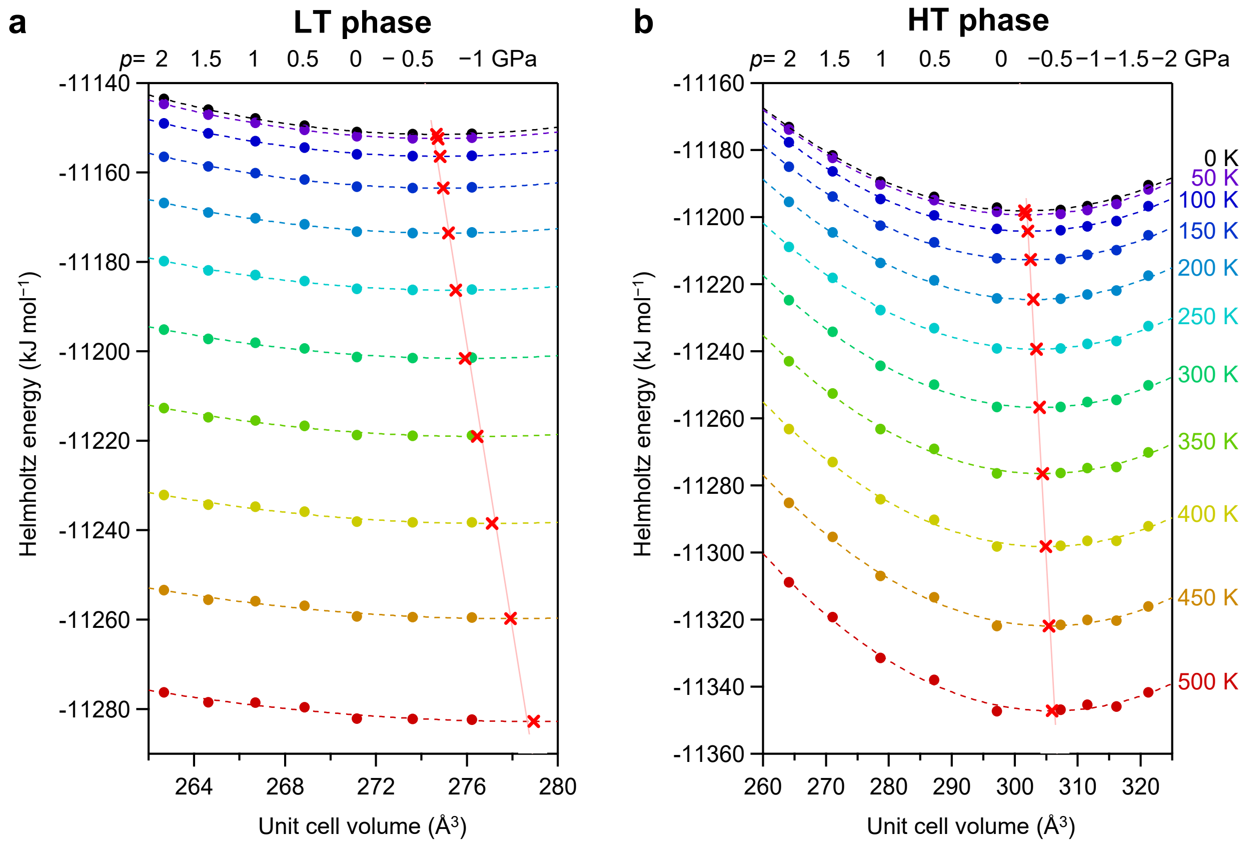

Supplementary Figure 16. Phonon mode calculation of the thermal expansion coefficient. Helmholtz energy versus unit cell volume at different temperatures for the LT phase (**a**) and HT phase (**b**). The unit cell was optimized at 9 different pressures, +2.0, +1.5, +1.0, +0.5, 0, −0.5, −1.0, −1.5, and −2.0 GPa.† Dotted curved lines and red crosses indicate the fitted curves and minimum energy values for each temperature, respectively. Pink lines passing through the crosses are the linear fitted lines corresponding to the thermal expansion coefficient.

† For the LT phase, −1.5 and −2.0 GPa conditions could not be calculated because the LT phase structure cannot be maintained.

§ 10. Measurement of temperature change of cyano-RbMnFeCo

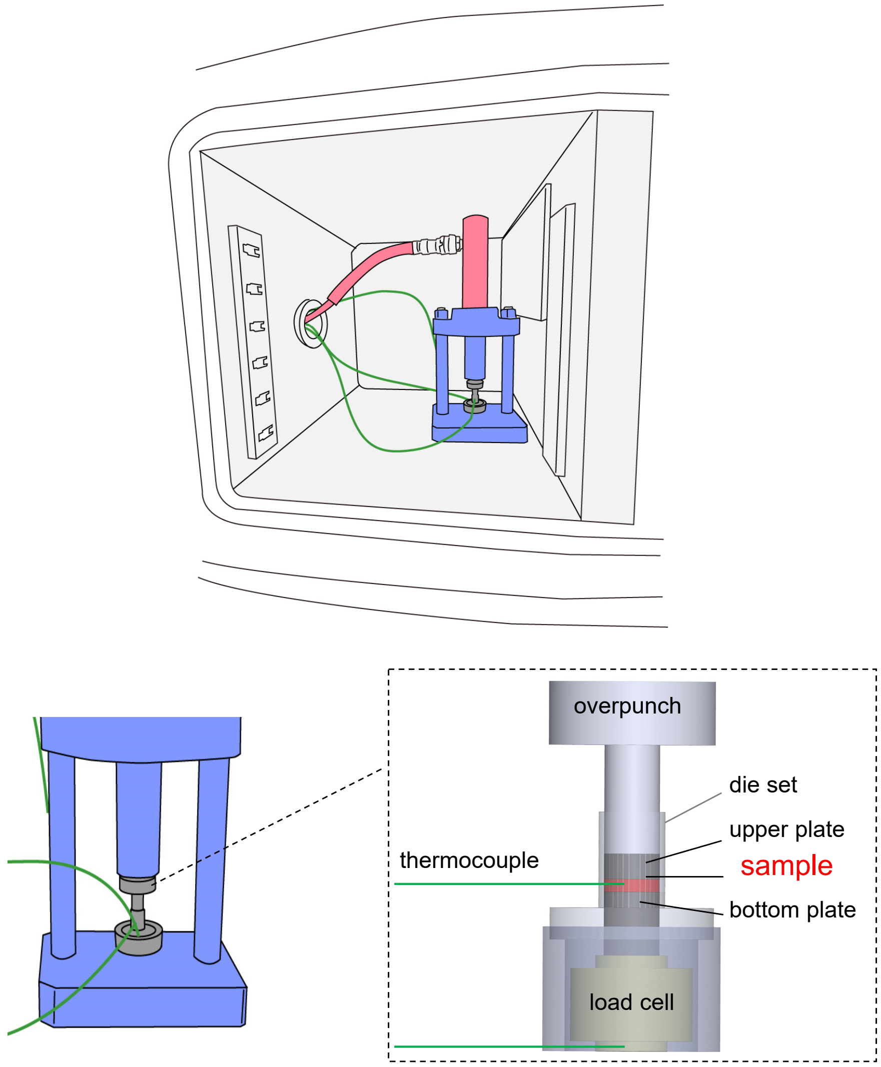

Supplementary Figure 17. Direct measurement system using a thermocouple for the temperature change (ΔT_{obs}) by applying and releasing pressure. We constructed an own-made system, which uses a thermocouple, to measure the temperature changes of a pellet-shaped sample while applying and releasing the uniaxial pressure (Supplementary Table 7). The pellet is composed of randomly oriented crystals.

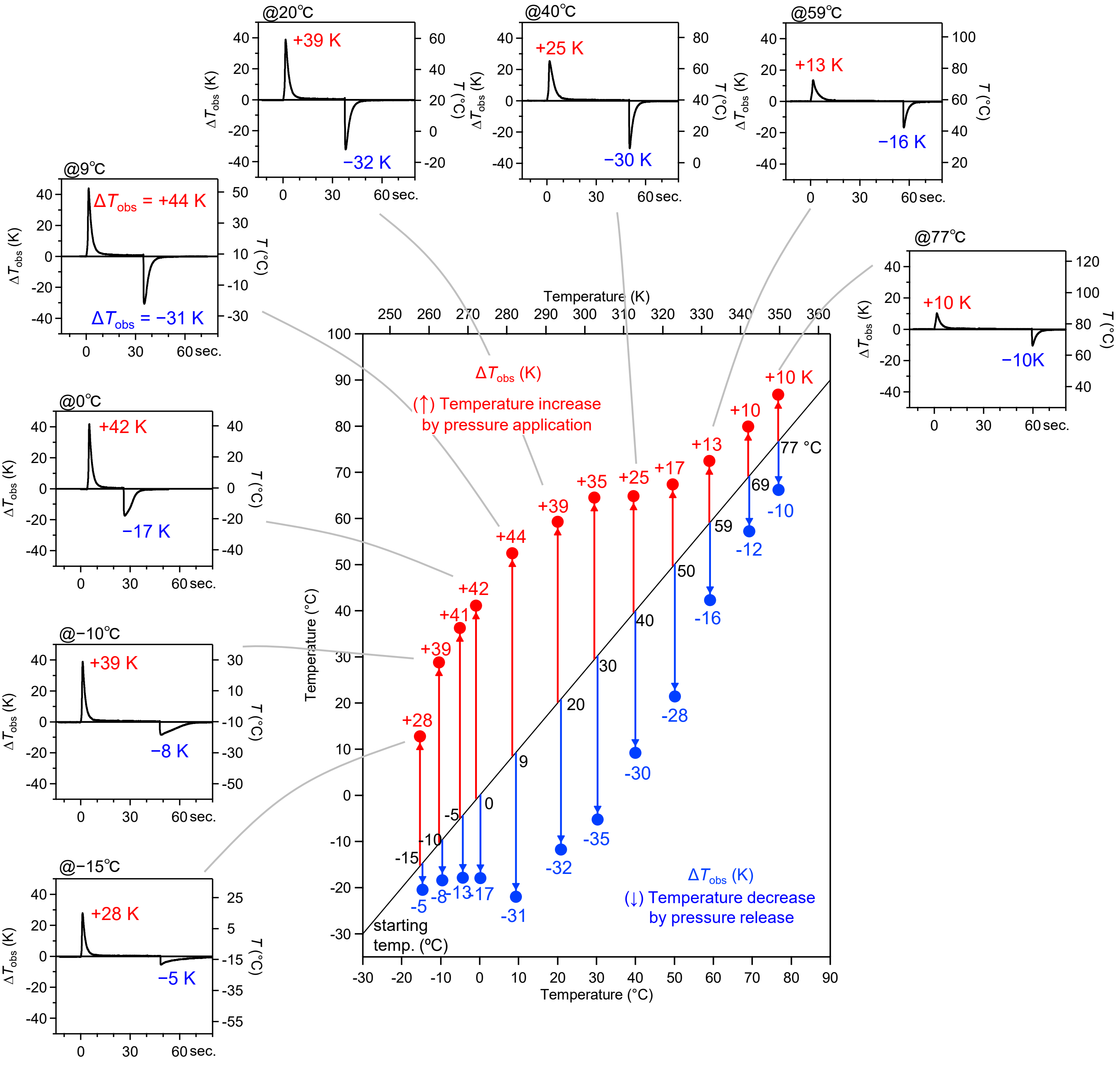

Supplementary Figure 18. Starting temperature dependence of ΔT_{obs}. Lower right figure shows the temperature increase (red) and decrease (blue) by applying and releasing pressure at different starting temperatures. Surrounding figures are the time dependences of ΔT_{obs} at starting temperatures of 77 °C, 59 °C, 40 °C, 20 °C, 9 °C, 0 °C, −10 °C, and −15 °C. The time decay of ΔT_{obs} depends on the starting temperature. In the future, we plan to improve the experimental setup for higher thermal insulation.

§ 11. Material cost

From the viewpoint of material costs for mass production, the present material meets the necessary conditions. Especially, the cost of rubidium in the stage of mass production is inexpensive as well as those of manganese and iron by the following reasons; (i) rubidium is an abundant element on the earth, (ii) low purity reagents of rubidium are applicable for the synthesis because rubidium is selectively adsorbed into the pores of the Mn–NC–Fe framework, and (iii) in the large-scale synthesis such as ton-scale production, the cost can be remarkably reduced. This information concerning the costs of the starting materials have been confirmed in the discussions with industrial companies.

§ 12. Devices and sensors for measurements

Supplementary Table 7. Devices and sensors, and their accuracies used for measurements.

Device / sensor	Manufacturer	Accuracy
SQUID magnetometer, MPMS	Quantum Design	Sensing coil accuracy 0.1%<br>Thermometer error 0.01%
PPMS	Quantum Design	Temperature accuracy ±0.5%
DSC (ambient pressure), DSC8230	Rigaku	Accuracy ±0.1%
High-pressure DSC, Calvet HT	Setaram	Temperature accuracy ±1 °C<br>Enthalpy accuracy ±1%<br>Calorimetric precision ±0.5%
Thermocouple, TC2-S316-T0.25Z100-2	Toyotech	Response time 0.012 s<br>Calibration for the temperature deviation by pressure: 0.101 ± 0.003 K per 100 MPa
Temperature sensor, LMC-A-50KN	Kyowa Electron. Instr.	Accuracy ±1% of rated output<br>Sampling frequency 2 kHz
Temperature chamber, MC-812R	ESPEC	Accuracy ±0.5 °C

§ 13. Supplementary References

1. Aznar, A. et al. Reversible colossal barocaloric effects near room temperature in 1-X-adamantane (X = Cl, Br) plastic crystals. *Appl. Mater. Today* **23**, 101023 (2021).
2. Aznar, A. et al. Reversible and irreversible colossal barocaloric effects in plastic crystals. *J. Mater. Chem. A* **8**, 639–647 (2020).
3. Romanini, M. et al. Giant and reversible barocaloric effect in trinuclear spin-crossover complex $Fe_3(bntrz)_6(tcnset)_6$. *Adv. Mater.* **33**, 2008076 (2021).
4. Ren, Q. et al. Ultrasensitive barocaloric material for room-temperature solid-state refrigeration. *Nat. Commun.* **13**, 2293 (2022).
5. Li, J. et al. Colossal reversible barocaloric effects in layered hybrid perovskite $(C_{10}H_{21}NH_3)_2MnCl_4$ under low pressure near room temperature. *Adv. Funct. Mater.* **31**, 2105154 (2021).
6. Lloveras, P. et al. Colossal barocaloric effects near room temperature in plastic crystals of neopentylglycol. *Nat. Commun.* **10**, 1803 (2019).
7. Imamura, W., Usuda, E. O., Lopes, É. S. N. & Carvalho, A. M. G. Giant barocaloric effects in natural graphite/polydimethylsiloxane rubber composites. *J. Mater. Sci.* **57**, 311–323 (2022).
8. Salvatori, A. et al. Large barocaloric effects in two novel ferroelectric molecular plastic crystals. *J. Mater. Chem. A* **11**, 12140–12150 (2023).
9. Imamura, W. et al. Supergiant Barocaloric Effects in Acetoxy Silicone Rubber over a Wide Temperature Range: Great Potential for Solid-state Cooling. *Chinese J. Polym. Sci.* **38**, 999–1005 (2020).
10. Aznar, A. et al. Giant barocaloric effects over a wide temperature range in superionic conductor AgI. *Nat. Commun.* **8**, 1851 (2017).
11. Li, J. et al. Reversible barocaloric effects over a large temperature span in fullerite C_{60}. *J. Mater. Chem. A* **8**, 20354–20362 (2020).
12. Seo, J. et al. Colossal barocaloric effects with ultralow hysteresis in two-dimensional metal–halide perovskites. *Nat. Commun.* **13**, 2536 (2022).
13. Parlinski, K. et al. First-principles determination of the soft mode in cubic ZrO_2. *Phys. Rev. Lett.* **78**, 4063−4066 (1997).
14. Tokoro, H. et al. Theoretical prediction of a charge-transfer phase transition. *Sci. Rep.* **8**, 63 (2018).
15. Yoshida, T. et al. Extremely low-frequency phonon material and its temperature- and photo-induced switching effects. *Chem. Sci.* **11**, 8989–8998 (2020).
16. MedeA 3.4; Materials Design, Inc., San Diego, USA.
17. Kresse, G. & Furthmüller, J. Efficient iterative schemes for ab initio total-energy calculations using a plane-wave basis set. *Phys. Rev. B* **54**, 11169–11186 (1996).
18. Kresse, G. & Furthmüller, J. Efficiency of ab-initio total energy calculations for metals and semiconductors using a plane-wave basis set. *Comput. Mat. Sci.* **6**, 15–50 (1996).
19. Kresse, G. & Joubert, D. From ultrasoft pseudopotentials to the projector augmented-wave method. *Phys. Rev.* **59**, 1758–1775 (1999).
