# Supplementary material for: Giant adiabatic temperature change and its direct measurement of a barocaloric effect in a charge-transfer solid
Source: Nat Commun. 2023 Dec 27;14:8466. doi: 10.1038/s41467-023-44350-4 (PMC10752886; doi:10.1038/s41467-023-44350-4)
Supplement: Supplementary file 3 — Description of Additional Supplementary Files [file 41467_2023_44350_MOESM3_ESM.pdf]

Description of Additional Supplementary Files

Supplementary Movie 1: Computer simulation of the reversible barocaloric effect of cyano-RbMnFeCo.

First, the calculated entropy curves for the HT and LT phases are shown on the left as $S_{HT}(T)$ and $S_{LT}(T)$, respectively. The gray-shaded area shows the hysteresis loop under atmospheric pressure. On the right, the crystal structure is shown, and then, a schematic illustration of a cooling system using **cyano-RbMnFeCo** appears. The next part shows the calculation of a reversible refrigeration cycle. Initially, the material is in the HT phase at the operation temperature. In the isothermal pressure application process, the thermal hysteresis shifts to higher temperatures and **cyano-RbMnFeCo** transforms from the HT phase to the LT phase at a constant temperature. Next, in the adiabatic pressure release process, the thermal hysteresis shifts to lower temperatures, and the LT phase returns to the HT phase at constant entropy. This is accompanied by a temperature decrease corresponding to the calculated reversible adiabatic temperature change ($|\Delta T_{ad,rev,calc}|$). Finally, the system comes into contact with the surroundings, and the material returns to the original HT phase through heat exchange. By repeating these three steps, the **cyano-RbMnFeCo** refrigerant can cool the surroundings.
